# Integrated RNA gene expression analysis identified potential immune-related biomarkers and RNA regulatory pathways of acute myocardial infarction

**DOI:** 10.1371/journal.pone.0264362

**Published:** 2022-03-01

**Authors:** Guangyao Shao

**Affiliations:** Qingdao University, Shan Dong province, Ri Zhao, China; University of Science and Technology Liaoning, CHINA

## Abstract

**Background:**

Acute lesions are among the most important causes of death due to vascular lesions worldwide. However, there are no accurate genetic markers for Acute myocardial infarction (AMI). This project will use microarray integration analysis in bioinformatics analysis to find and validate relevant AMI gene markers.

**Methods:**

Five microarray gene expression datasets were downloaded through the GEO database. We identified 50 significant DEGs by comparing and analyzing gene expression between 92 AMI and 57 standard samples. The BioGPS database screened differentially expressed genes specific to the immune system. DEGs were mainly involved in immune-related biological processes based on Enrichment analysis. Eight hub genes and three-gene cluster modules were subsequently screened using Cytoscape and validated using Box plot’s grouping comparison and ROC curves. Combined group comparison results and ROC curves analysis concluded that *AQP9*, *IL1B*, and *IL1RN* might be potential gene markers for the AMI process. We used the StarBase database to predict target miRNAs for eight essential genes. The expected results were used to screen and obtain target lncRNAs. Then Cytoscape was used to create CeRNA networks. By searching the literature in PubMed, we concluded that *AQP9*, *IL1B*, and *IL1RN* could be used as gene markers for AMI, while *FSTL3-miR3303p-IL1B/IL1RN* and *ACSL4-miR5905p-IL1B* could be used as RNA regulatory pathways affecting AMI disease progression.

**Conclusions:**

Our study identified three genes that may be potential genetic markers for AMI’s early diagnosis and treatment. In addition, we suggest that *FSTL3-miR-330-3p-IL1B/IL1RN* and *ACSL4-miR-590-5p-IL1B* may be possible RNA regulatory pathways to control AMI disease progression.

## Introduction

Acute myocardial infarction (AMI) is a common cardiovascular disease and is one of the most prevalent cardiovascular diseases. Acute myocardial infarction strongly correlates with the immune system [1]. Acute myocardial infarction is associated with inflammation of the patient’s epicardial coronary arteries. It has been shown that plaque rupture is related to systemic inflammation and activation of inflammatory signals from macrophages in the local plaque. For example, damage-associated proteins (DAMPs) are released during the post-infarct phase of the inflammatory response and stimulate the body’s immune response. During this phase, leukocytes are mobilized, and neutrophils, monocytes, and lymphocytes wander into the infarcted area. Later, pro-inflammatory signals are suppressed, and leukocyte infiltration is terminated. The inflammatory and immune response eventually causes irreparable damage to the myocardial cells and places a heavy burden on them [2]. Therefore, early diagnosis and treatment of acute myocardial infarction (AMI) can prevent disease progression.

Transcriptome and microarray analysis are becoming more common in AMI studies [3–5]. Studies have shown that the LncRNA *SNHG8* regulates acute myocardial infarction [6]. However, there are no validated genetic markers for acute myocardial infarction at the transcriptome level. Finding new genetic markers at the transcriptome level is crucial for early detection and effective treatment of AMI. In addition, there are many studies applying bioinformatics methods to study biological markers of acute myocardial infarction, such as weighted gene co-expression network analysis [7], integrated bioinformatics analysis, etc. [8]. However, these studies did not combine genomes into a gene matrix for integrated analysis. This search for potential mRNAs is achievable through integrated analysis of microarrays in bioinformatics.

Five microarray datasets from the GEO database were downloaded for the current study. Robust Multiarray Average (RMA) in R was used to normalize all array data. DEGs were screened using screening criteria (P<0.05, LogFC>1.5). The online program BioGPS was used to screen genes expressed explicitly by the immune system for our study. The R software cluster analysis package was used to perform enrichment analysis on the GO and KEGG databases. We constructed a PPI network using the STRING database (https://string-db.org/) and the Cytoscape plugins MCODE and cytohubba. We obtained eight central genes expressed explicitly by the immune system by extracting and analyzing three cluster modules in the PPI network. After that, we validated the acquired essential genes by group comparison and ROC curves using the integrated GEO dataset. We used StarBase (version 3.0) (http://starbase.sysu.edu.cn/index.php) to predict the target miRNAs of the eight essential genes. Long non-coding RNAs regulating miRNAs were further obtained based on the above miRNA prediction results. Cytoscape was used to build a CeRNA network of interrelationships between miRNAs lncRNAs and critical genes. To validate the potential gene markers, we conducted a literature search. Finally, *AQP9*, *IL1B*, and *IL1RN* were identified as potential key genes involved in AMI. In addition, we also investigated the RNA regulatory routes of *AQP9*, *IL1B*, and *IL1RN*. This study provided us with new insights into the complex immune process of AMI at the transcriptome level and identified potential gene markers and associated RNA regulatory pathways for the diagnosis of AMI.

## Materials and methods

### Analysis workflow

**Fig 1:**
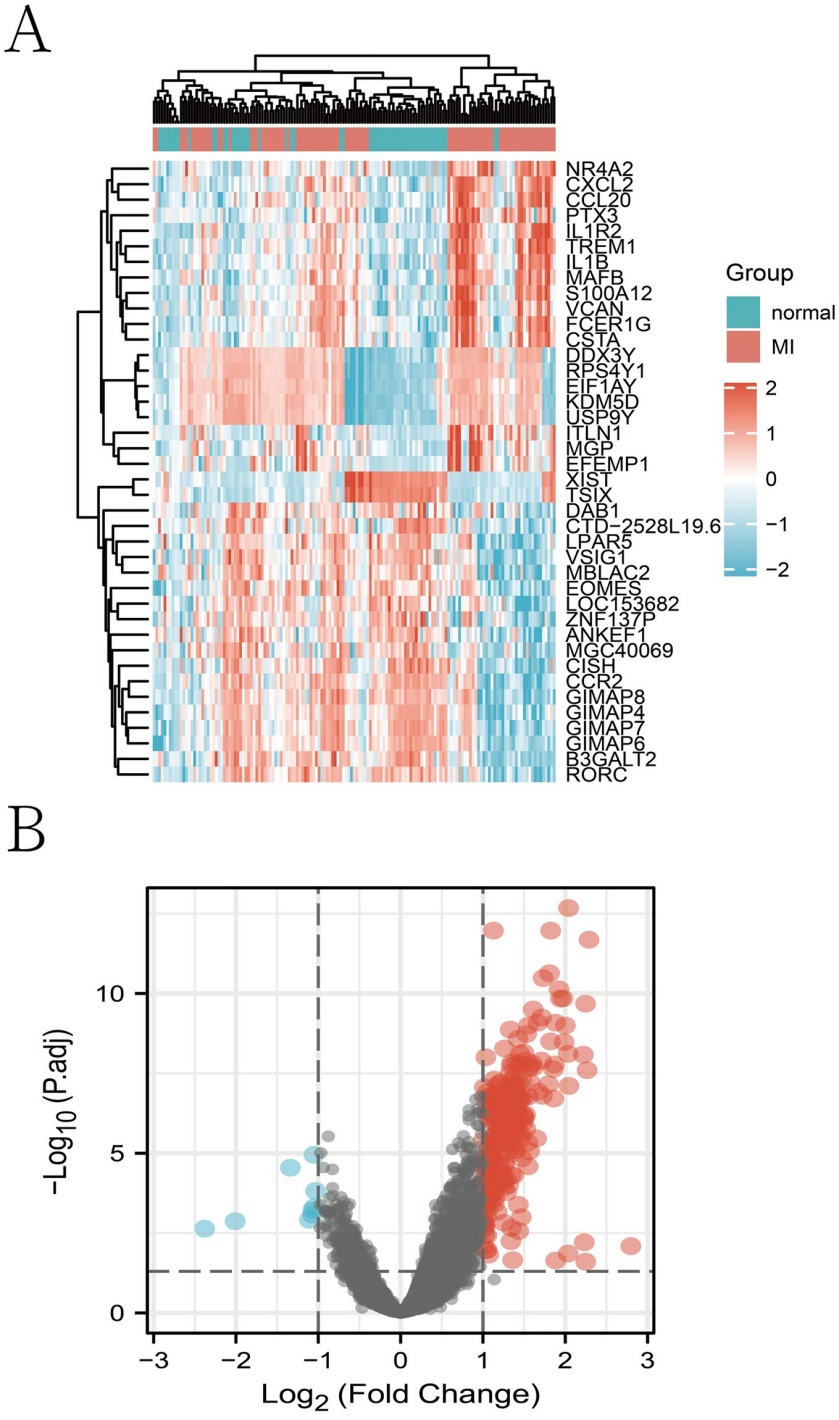
**A. Heat map of gene expression**. Heat map of gene expression with expression levels shown as rectangles of different colors: red indicates high levels of differential genes, while Combined with the relationship between their expression levels blue indicates low levels of differential genes. **B. Vulcano plot**. Vulcano plot showing the difference in DEGs between AMI samples and healthy controls. Genes that are overexpressed are shown in red, while temporarily unrelated genes are shown in black. Genes that are downregulated are shown in blue.

The analytical workflow diagram for this study is shown in Fig 1. We downloaded data from the GEO database and divided them into two groups(The test and validation sets). The test set was analyzed after quality control. Fifty differential genes were screened according to the screening conditions and enriched for analysis. We then combined the 50 screened differential genes with the PPI network and BIOGPS database to obtain eight essential genes. We validated these eight critical genes in the validation set data. Finally, we predicted the miRNAs and lncRNAs of these three hub genes, which led to two regulatory pathways of AMI.

### Data acquisition

The data was collected using the GEO database. The following were used as screening criteria. (1) array data obtained using Homo sapiens gene expression analysis; (2) whole blood and endothelial cells; (3) files with sample-related information in the data. (4) No duplicates were analyzed for each subject. GSE29111, GSE19339, GSE97320, and GSE66360, including 92 AMI samples and 57 standard samples, were merged after QC (quality control), and the combined dataset was filtered again. They were selected as the test set for differentially expressed gene analysis and the validation set for group comparisons between immune system-associated Hub gene expression. GSE48060, including 31 AMI and 21 standard samples, was selected as the validation set for resistant system-associated Hub gene ROC curves (Table 1).

**Table 1:** Data set information summary.

GEO ID	Platform	Samples number		Source	Age		Sex (male/female)		Attribute (Test set/ Validation set)
		AMI	normal		AMI	normal	AMI	normal	
GSE29111	GPL570	36	0	whole blood	56.4±9.2	-	30/6	-	Both
GSE19339	GPL570	4	4	blood	-	-	-	-	Both
GSE97320	GPL570	3	3	blood	53±13	53.7±4.7	-	-	Both
GSE66360	GPL570	49	50	Endothelial Cells	-	-	-	-	Both
GSE48060	GPL570	31	21	blood	-	-	-	-	Set of validations

GPL570: [HG-U133 Plus 2] Human Genome U133 Plus 2.0 Affymetrix Array. "-" indicates that the user did not get any information.

### Data normalization and identification of *DEGs*

After QC (quality control), the raw mRNA microarray data obtained from the GEO database were merged, and the combined dataset was filtered. The merged dataset was then normalized by the R affy package using a robust multi-array average normalization method. The R package "limma" was used to perform differential genetic analysis between samples. After obtaining P values, multiple hypothesis testing and correction of multiple hypothesis testing were performed. P-value thresholds were determined based on the false discovery rate. The meaning of the corrected P-value is the adjusted P-value [9, 10]. Log2 (fold change) >1.5 or -1.5 and a P value of 0.05 were used as screening criteria.

### Heatmap and volcano plot analyses

Heatmaps and volcano plots were created using R software to illustrate the DEGs better. R’s heatmap package was used to create heatmaps.

### Identification of immune system-specific expressed genes

Use the online database BIOGPS (http://biogps.org/) [11] to screen for system-specific expression of genes in DEGs. The following were selected as screening criteria. (1) transcripts with expression values >10-fold the median associated with a single organ/system, and (2) expression values in the second-ranked system with only one-third of the expression of the first system [12]. Genes identified using these criteria were considered to be system-specific.

### Enrichment analysis

The R software clusterProfiler package was used in functional enrichment studies such as Gene Ontology (GO) annotation and Kyoto Encyclopedia of Genes and Genomes (KEGG) pathway enrichment analysis [13, 14]. P values less than 0.05 were used to identify significantly enriched functions and pathways.

To perform a more accurate enrichment analysis, we used GSEA analysis. This was done by using the clusterProfiler package for GSEA analysis. The selected datasets were BioCarta, KEGG, NABA, Reactome, WP [15, 16].

### Construction of the PPI network

A PPI network of DEGs with a total score more significant than 0.4 was built with STRING (https://string-db.org/). The Cytoscape program displayed the PPI network after obtaining interaction relationships. The MCODE (Minimal Common Oncology Data Elements) plugin [17] was used to identify significant gene clusters and cluster scores. Use Cytoscape plugin CytoHubbato to find critical genes in the PPI network [18]. A combination of five methods in the plugin CytoHubba was used to identify hub genes, including EPC (edge percolation component), MCC (leading edge centrality), MNC (maximum neighbour component), Degree (node connectivity), and Closeness (node connectivity tightness) [19, 20]. A comprehensive analysis of all the data led to the conclusions, then used to identify critical genes related to the immune system.

### Verification of the hub genes

GSE48060 (5 patients with event sample,26 patients without event sample,21 normal sample)was used to test these eight immune system-specific vital genes. Statistical analysis of the Student’s t-test and group comparison plots were performed using the R software ggplot2 and ggpubr packages. Five essential genes were expressed at higher levels in MI samples than in standard samples (P 0.01). *AQP9*, *IL1B*, and *IL1RN* were represented in the AMI samples above the standard samples (P < 0.05).

### *ROC* curve of the hub genes

AUC is a common method used to calculate the area under the *ROC* curve, representing the probability that a classifier will rank a randomly selected positive instance above a selected negative instance. It is computed as a trapezoidal calculation [21]. In this study, we use the pROC package for *ROC* analysis and the ggplot2 package for visualization [22]. For the GSE48060 dataset, we used R language to construct *ROC* curves based on the expression of eight immune system-specific key genes. We combined the patient with event and patient without event in GSE48060 as the AMI patient group, and the normal group was defined as the control group. And we performed ROC analysis with the pROC package. The area can describe the intrinsic efficacy of the diagnostic model under the curve (*AUC*) that combines sensitivity and specificity. These eight highly expressed key genes have better diagnostic value in AMI samples compared to normal samples. For example, *IL1RN* had the most incredible sensitivity and specificity in this cohort (AUC: 0.791), as well as S100A12 (*AUC*: 0.780), CCL4 (*AUC*: 0.780), S100A9 (*AUC*: 0.757), AQP9 (*AUC*: 0.740), and FPR1 (*AUC*: 0.694). Based on our current data, we predict that *AQP9*, *IL1B*, and *IL1RN* may be effective genetic markers for detecting ischemic stroke.

### Prediction of target miRNAs

Mutations and dysregulation of miRNAs and lncRNAs are inextricably linked to acute myocardial infarction [23, 24]. There are many models available to predict the relationship between miRNAs and LncRNAs. For example, the models used to predict the interrelationship between miRNA and disease, such as RWRMDA, HDMP, RKNNMDA, IMCMDA, etc. [25]. There are also models for predicting the interrelationship between miRNA and mRNA, such as Miranda, TargetScan, PicTar, etc. [24]. And models for predicting the relationship between lncRNAs and diseases, such as LNCSIM, LRLSLDA, ILNCSIM, RWRlncD, etc. [23]. and models for predicting the relationship between miRNAs and lncRNAs, such as LMI-INGI, EPLMI, LMFNRLMI, NDALMA, etc. [26, 27]. In addition, new algorithms are constantly being discovered, and please refer to the relevant literature for details [28–30]. We predicted hub gene target miRNAs using the online miRNA database StarBase(version 3.0; http://starbase.sysu.edu.cn/index.php) [31]. Cytoscape was used to build the mRNA-miRNA co-expression network. We sequentially input the 8 key genes into the miRNA prediction module of the starbase database and obtained the upstream miRNAs of the eight key genes. To make the prediction results more accurate, we defined the miRNAs that were judged as valid miRNAs by three or more methods in the starbase database results to be considered the key genes.

### Construction of ceRNA networks

Based on the interaction relationships between known miRNAs, we made predictions in StarBase (version 3.0) [31] to screen out lncRNAs that interact with known miRNAs. After that, Cytoscape was used to build a CeRNA network based on the linkage between mRNAs, miRNAs, and lncRNAs.

### Statistics analysis

We performed statistical analyses using the rgpubr R package. The R package for ggplot2 was used to create boxplots. we used the Student’s t-test to see any differences between the two groups. ROC analysis and ROC curves were performed using the ROCR program.

## Results

### Identification of *DEGs*

After QC (quality control), we merged the data set from 92 AMI and 57 standard samples and filtered the data. After performing differential gene analysis, we obtained two down-regulated genes and 48 others that were upregulated, giving us a total of 50 DEGs. Heat maps and volcano maps created using the R program can be seen in Fig 1.

### Identification of immune system-specific expressed genes

Fifty genes expressed in the organ/system were screened using BioGPS. These genes were found in the most significant proportion of the immune system. The second most represented tissue or organ system was the heart (11/50, 22%). This was followed by the digestive system (10/50, 20%). Finally, the nervous system, respiratory system, urinary system, coagulation system, skeletal, endocrine system, and skeletal muscle had the lowest percentage of genes (6/50, 12%) (Table 2).

**Table 2:** Summary of gene information.

System	Gene
Nerve system	CLEC4E TP53INP2 CD36 FAM198B IL1RN PITX3 XIST USP9Y;
Heart	THBD FN1 PLAUR SULF1 TP53INP2 CD36 EFEMP1 FAM198B IL1RN PITX3 THBS1
Skeletal muscle	TP53INP2 CD36 FAM198B IL1RN PITX3
Urinary system	NR4A2 TP53INP2 CD36 FAM198B IL1RN PITX3 KCNJ15
Immune system	THBD TP53INP2 PITX3 MAFB AQP9 CCL4 PLAUR C5AR1 HCAR3 NFIL3 S100A9 RPS4Y1 TREM1 CSF3R KDM5D IL1R2 CD36 IL1B FCER1G FAM198B CD83 IL1RN S100A12 FPR1
Coagulation system	TP53INP2 ITLN1 CD36 FAM198B IL1RN PITX3
Bone	IL1R2 CLEC4E FCER1G CD36 FAM198B IL1RN PITX3
Digestive system	THBD C15orf48 MAFB ACSL1 ITLN1 SERPINA1 CD36 FAM198B IL1RN PITX3
Endocrine System	CLEC4E TP53INP2 MGP CD36 FAM198B IL1RN PITX3 FN1
Respiratory system	THBD CXCL16 ITLN1 CCL20 IER3 CD36 FAM198B IL1RN PITX3

Shows the correlation of genes with different systems

### Enrichment analysis

**Fig 2:**
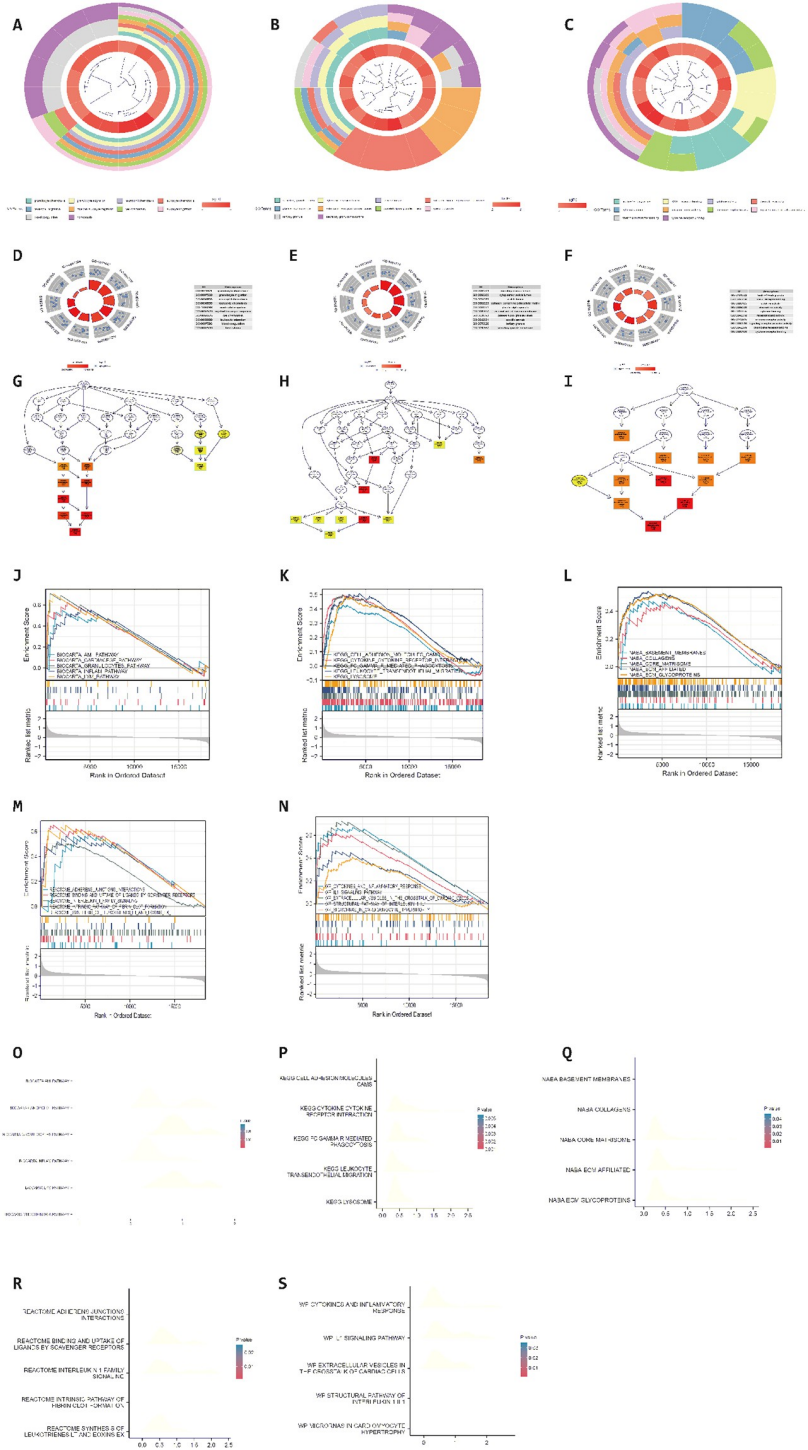
**A, GO Circle plot of biological process**. The GO circle plot of biological processes shows the biological processes of enrichment. The main enrichment processes are leukocyte chemotaxis:. **B, GO Circle plot of cellular component**. The GO circle plot of cellular constituents shows the enriched cellular component. The main enriched cellular component is collagen-containing extracellular matrix. **C, GO Circle plot of molecular function**. GO circles plot of molecular functions shows the enriched molecular functions. The main enriched molecular function is chemokine activity. **D. GO Circle of biological process**. GO circle of biological processes shows the enriched biological processes. The main enriched processes are granulocyte chemotaxis, granulocyte migration, neutrophil chemotaxis, leukocyte chemotaxis, neutrophil migration, myeloid leukocyte migration, cell chemotaxis, leukocyte migration, blood coagulation, hemostasis. **E. GO Circle of cellular component**, The GO circle of cellular components shows the enriched cellular components. The main enriched cellular components are secretory granule lumen, cytoplasmic vesicle lumen, vesicle lumen, collagen–containing extracellular matrix, platelet alpha granule, external side of plasma membrane, platelet alpha granule lumen, specific granule, tertiary granule, secretory granule membrane. **F. GO Circle of molecular function**, The GO circle of molecular function shows the enriched molecular function. The main enriched molecular functions are ficolin-1-rich granule, RAGE receptor binding, cytokine activity, chemokine activity, cytokine binding, receptor-ligand activity, immune receptor activity, signaling receptor activator activity, chemokine receptor binding, cytokine receptor binding. **G. GO Functional enrichment network diagram of biological process**. The GO functional enrichment network diagram of biological processes shows the enriched biological processes. The main enrichment process is neutrophil chemotaxis. **H. GO Functional enrichment network diagram of cellular component**. The GO functional enrichment network diagram for cellular components shows the enriched cellular components. The main enriched cellular component is platelet alpha granule lumen. **I. GO Functional enrichment network diagram of molecular function**. The GO functional enrichment network diagram for molecular functions shows the enriched molecular functions. The main enriched molecular function is chemokine activity. **J. GSEA Enrichment plot of BioCarta gene sets**. GSEA Enrichment plot of BioCarta gene sets showing the pathway enrichment of 50 differential genes in the BioCarta genome. The main enriched pathways are AMI PATHWAY, CARDIACEGF PATHWAY, GRANULOCYTES PATHWAY, INFLAM PATHWAY, LYM PATHWAY. **K. GSEA Enrichment plot of KEGG gene sets**. The pathway enrichment of 50 differential genes in the KEGG genome is shown. The main enriched pathways are CELL ADHESION MOLECULES CAMS, CYTOKINE RECEPTOR INTERACTION, FC GAMMA R MEDIATED PHAGOCYTOSIS, LEUKOCYTE TRANSENDOTHELIAL MIGRATION, LYSOSOME. **L. GSEA Enrichment plot of NABA gene set**. GSEA Enrichment plot of NABA gene set shows the enrichment of the cellular components of the 50 differential genes in the NABA genome. The main enriched components are BASEMENT MEMBRANES, COLLAGENS, CORE MATRISOME. ECM AFFILIATED, ECM GLYCOPROTEINS. **M. GSEA Enrichment plot of Reactome gene sets**. GSEA Enrichment plot of the Reactome genome showing the functional enrichment of 50 differential genes in the Reactome genome. The main enriched functions are ADHERENS JUNCTIONS INTERACTIONS, BINDING, AND UPTAKE OF LIGANDS BY SCAVENGER RECEPTORS, INTERLEUKIN 1 FAMILY SIGNALING, INTRINSIC PATHWAY OF FIBRIN CLOT FORMATION, SYNTHESIS OF LEUKOTRIENES LT AND EOXINS EX. **N. GSEA Enrichment plot of WP Gene Set**. The enrichment plot of WP Gene Set shows the pathway enrichment of 50 differential genes in the WP genome. The main enriched pathways are CYTOKINES AND INFLAMMATORY RESPONSE, IL1 SIGNALING PATHWAY, EXTRACELLULAR VESICLES IN THE CROSSTALK OF CARDIAC CELLS, STRUCTURAL PATHWAY OF INTERLEUKIN 1 IL1, MICRORNAS IN CARDIOMYOCYTE HYPERTROPHY. **O. GSEA ridge plot of BioCarta gene sets**. That Shows the pathway enrichment of 50 differential genes in the BioCarta genome. **P. GSEA ridge plot of KEGG gene sets**. The pathway enrichment of the 50 differential genes in the KEGG genome is shown. **Q. GSEA ridge plot of NABA gene set**. That Shows the cellular component enrichment of the 50 differential genes in the NABA genome. **R. GSEA ridge plot of Reactome gene sets**. The functional enrichment of the 50 differential genes in the Reactome genome is shown. **S. GSEA ridge plot of WP Gene Set**. That Shows the pathway enrichment of the 50 differential genes in the WP genome.

The Gene Ontology (GO) plot shows the functions and pathways of gene enrichment. The results show that immune response, immune-related chemotaxis, has the most significant enrichment in terms of gene function. GSEA showed genes closely associated with AMI PATHWAY, CARDIACEGF PATHWAY, etc. The screening thresholds are p 0.05 and Q 0.05. NES stands for the normalized enrichment score, and p.adjust means to change the p-value (Fig 2).

### Construction of the PPI network

**Fig 3:**
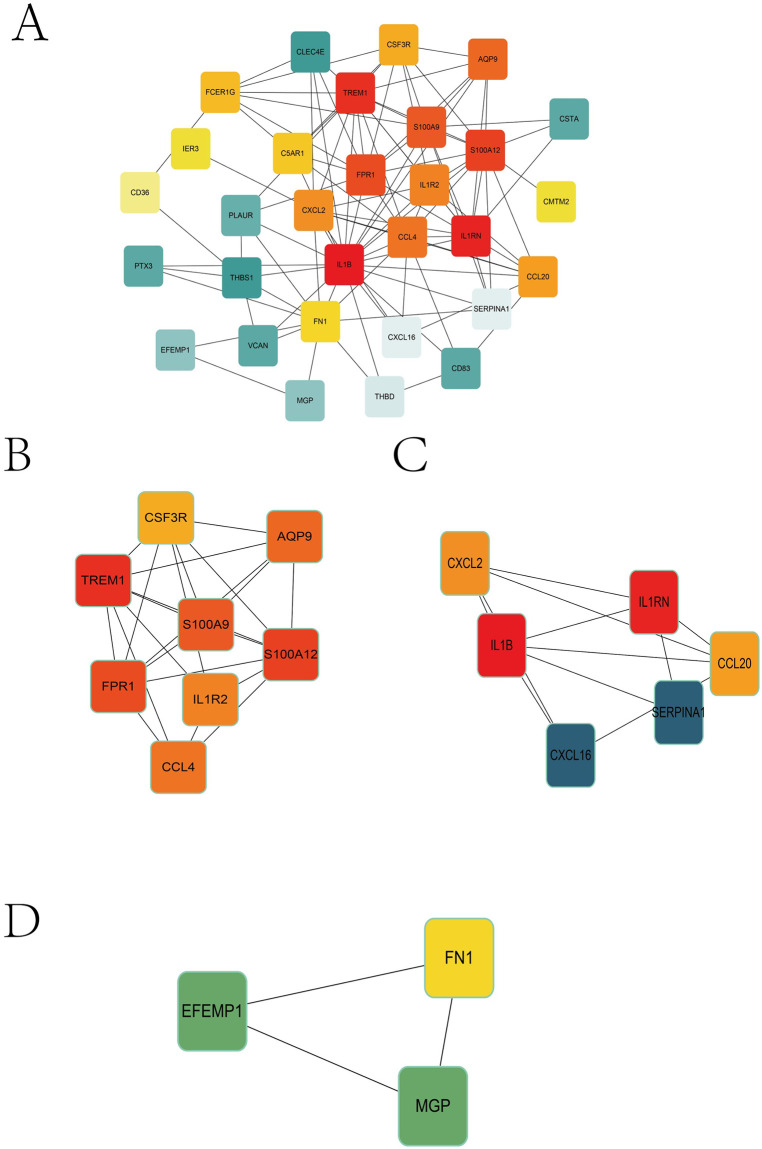
PPI network. MCODE uncovered a DEG PPI network with three cluster components. a Protein-protein interaction network generated by DEGs. Protein-protein associations are represented by the nodes and edges of the graph. Red diamonds express upregulated genes, whereas green and blue hexagons represent downregulated genes. MCODE was able to extract three cluster modules. With the most points, Cluster 1 (b) was the winner, followed by Cluster 2 (c) and Cluster 3. (d).

The PPI network generated with STRING demonstrates the interaction relationships between the proteins encoded by DEGs. The cytoHubba plugin was used to determine the importance of genes in the genome. We identified eight immune system-associated Hub genes using at least five cytohubba target prediction tools. These genes scored high in cytohubba’s five target prediction techniques and are critical for understanding the pathogenesis of AMI (Fig 3).

### Verification of the hub genes

**Fig 4:**
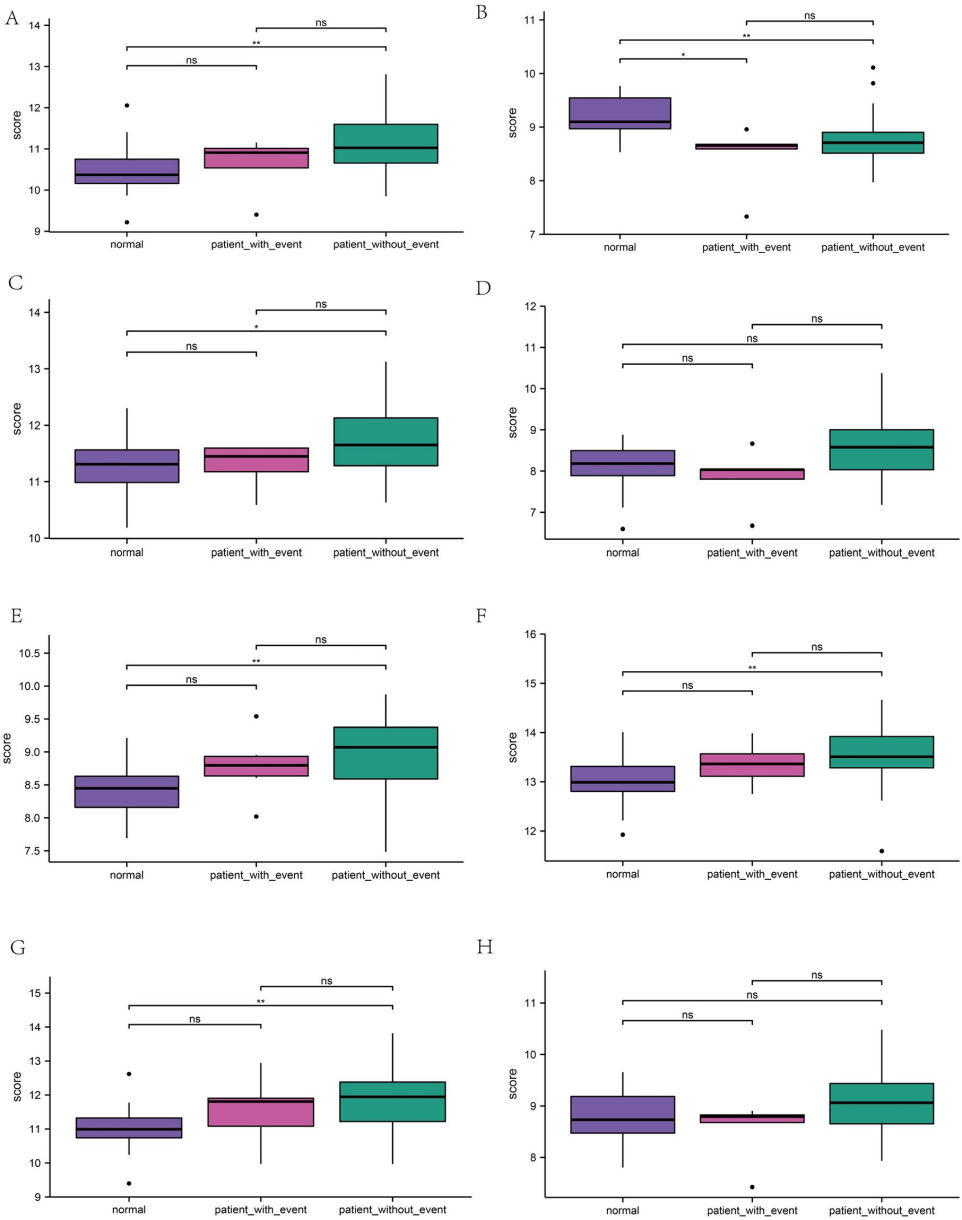
Box plots for group comparisons. Verification of the eight immune system-specific hub genes that are expressed. ***: p < 0.001, **: p < 0.01, *: p < 0.05, ns: no significant difference. A significant upregulation of five genes occurs in MI samples compared to standard samples. **A. Group comparisons of *AQP9*. B. Group comparisons of *CCL4*. C. Group comparisons of *FPR1*. D. Group comparisons of *IL1B*. E. Group comparisons of *IL1RN*. F. Group comparisons of *S100A9*. G. Group comparisons of *S100A12*. H. Group comparisons of *TREM1***.

To validate the eight immune system-specific hub genes obtained in the GSE48060, we performed Student’s t-test statistics and constructed boxplots using the ggplot2 and ggpubr software packages for R. The expression levels of the five hub genes were significantly higher in the MI samples compared to the standard samples (P 0.01) (Fig 4). expression levels of *AQP9*, *IL1B*, and *IL1RN* were substantially higher in the MI samples compared to the standard samples (P 0.05).

### *ROC* curve of the hub genes

**Fig 5:**
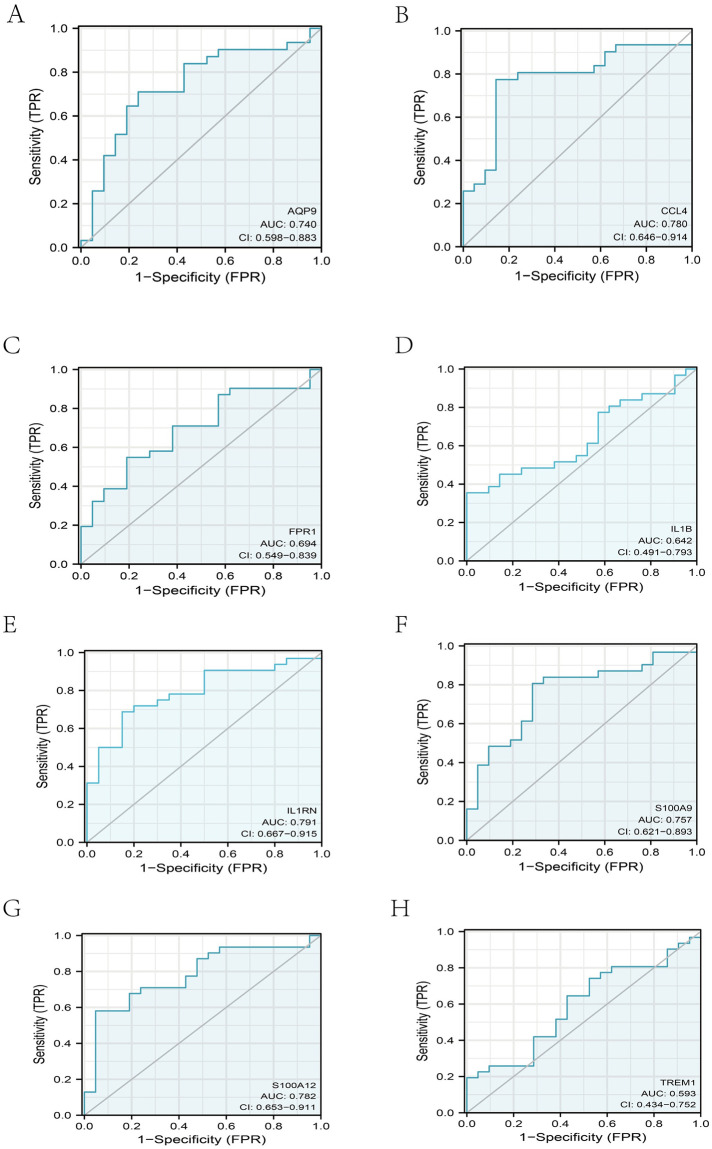
ROC plot. Gene expression ROC curve for the eight immune system-specific hub genes. AUC: the surface area under the ROC curve **A. ROC plot of *AQP9*. B. ROC plot of *CCL4*. C. ROC plot of *FPR1*. D. ROC plot of *IL1B*. E. ROC plot of *IL1RN*. F. ROC plot of *S100A9*. G. ROC plot of *S100A12*. H. ROC plot of *TREM1***.

For the GSE48060 dataset, we analyzed eight immune system-specific expressions of key genes using R and constructed *ROC* curves. The area measured the overall validity and sensitivity of the diagnostic test under the curve (*AUC*) [32]. Il1RN had the best diagnostic value of all these genes (*AUC*: 0.791). Others with diagnostic significance included S100A12 (*AUC*: 0.782), CCL4 (*AUC*: 0.781) S100A9 (0.757), AQP9 (0.750), FPR1 (0.750), IL1B (0.640), and TREM1 (0.593) (Fig 5). We combined these results with the expression levels of these genes in health and MI-related samples to identify potentially valid gene markers. Our current samples lead us to believe that the biomarkers *AQP9*, *IL1B*, and *IL1RN* may contribute to the early detection of AMI.

### Prediction of target miRNAs

**Fig 6:**
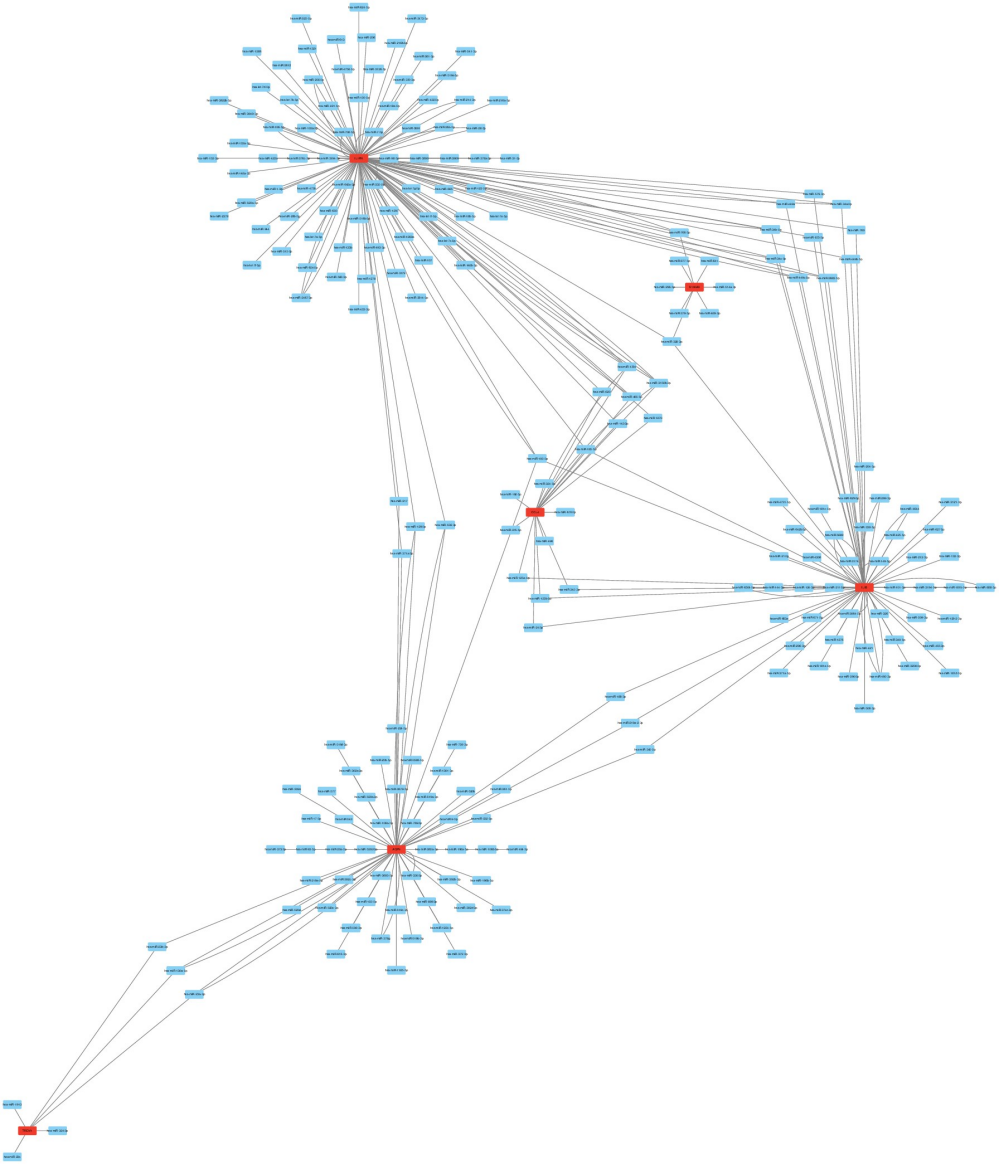
The mRNA-miRNA co-expression network. The mRNA-miRNA co-expression network was designed using Cytoscape. In a network, hub genes and miRNA targets are co-expressed together. TREM1 contains just six target miRNAs, compared to IL1RN’s dozens of miRNAs. The nodes represent mRNA and miRNA, while the edges represent the relationship between mRNA and miRNA interaction.

According to our method’s definition of valid miRNAs, we screened five genes containing valid miRNAs from 8 essential genes. We predicted target miRNAs for hub genes with five different miRNA databases. We obtained 293 target miRNAs from eight highly expressed hub genes. Based on the predicted results, Cytoscape created a hub gene and miRNA co-expression network (Fig 6).

### Construction of ceRNA networks

**Fig 7:**
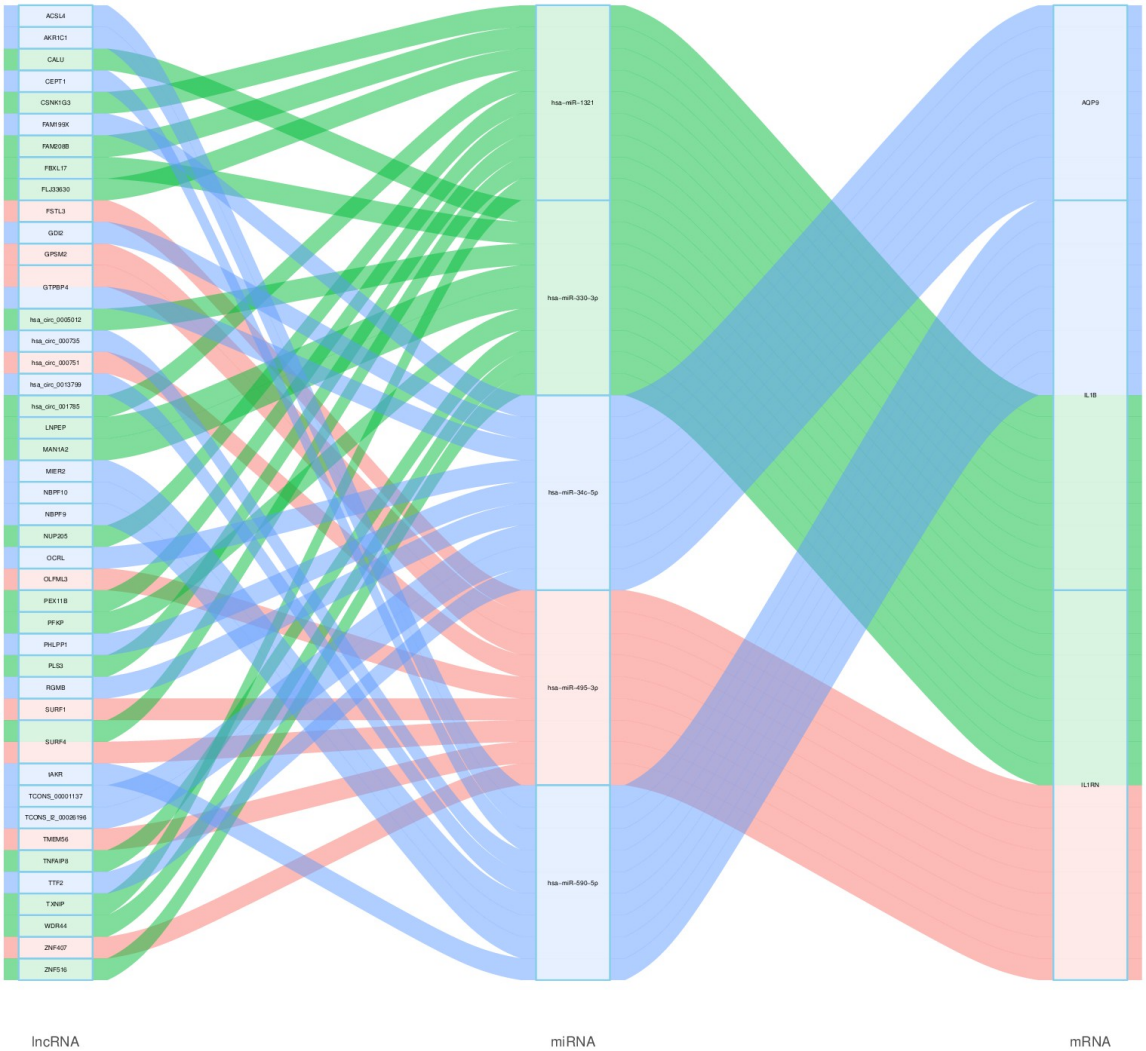
ceRNA networks. Three ceRNA networks comprised *AQP9*, *IL1B*, and *IL1RN*, as well as possible RNA regulatory mechanisms.

MiRNAs can silence genes and reduce gene expression through binding to mRNAs. miRNA function may be influenced by upstream molecules (lncRNAs) that bind miRNA response elements and thus upregulate gene expression [33]. We used CLIP-Data with high stringency (> = 5) in the human h19 genome in Starbase 3.0 as well as degradome data to predict upstream molecules (lncRNAs). There are several miRNA shear sites in one transcript. Therefore, we selected the upstream molecules (lncRNAs) with the highest scores in the Starbase database as the upstream target molecules (lncRNAs). Subsequently, we identified one target miRNA, nine *IL1B* target lncRNAs for *AQP9*, and two target miRNAs, and 14 *IL1B* target lncRNAs for *IL1RN*. ceRNA networks are demonstrations of the interaction relationships between RNAs. We created the ceRNA network using Cytoscape based on the prediction results. We screened one down-regulated miRNA and two upregulated lncRNAs reported in studies for further analysis based on the literature search. These RNA regulatory mechanisms may help to control the course of AMI by suppressing the expression of pro-inflammatory cytokines such as *IL1B/IL1RN* and *IL1B* (Fig 7).

## Discussion

Pathogenesis of AMI differs from patient to patient. Cardiomyocyte necrosis may be prevented, and the quality of life improved for patients with early diagnosis and treatment of AMI. AMI, on the other hand, still lacks reliable biomarkers. The discovery of novel and efficient genetic biomarkers for the early detection and treatment of AMI is critical.

We finalized 50 DEGs by comparing gene expression in AMI and standard samples.GO enrichment analysis of DEGs showed that differential genes were mainly enriched in immune cell migration, especially chemotaxis, and regulation of humoral immune responses.KEGG analysis was enriched primarily on the AMI pathway, CARDIACEGF pathway, etc.

After screening and validating the immune-related central genes by the PPI network established by Cytoscape, we identified eight genes differentially expressed in the immune system. ROC analysis is a method used to test the validity of a specific gene as a classifier and its function.*ROC* curve analysis showed that all of these genes were statistically significant. Combined with the relationship between their expression levels in AMI and the progression of the disease. Significant genes should be expressed at higher levels in patient samples than in normal samples.*AQP9*, *IL1B*, and *IL1RN* were consistent with statistical significance (p < 0.05). Therefore, it is reasonable to assume that *AQP9*, *IL1B*, and *IL1RN* may be important genetic biomarkers for diagnosing AMI. In addition, we also constructed a lncRNA-mRNA-miRNA co-expression network to elucidate the pathogenesis of AMI at the transcriptome level.

*AQP9*, a member of the cell membrane protein family, mainly transports water down the concentration gradient. An experimental animal study reported that this study demonstrated by qRT-PCR and Western blot experiments that silencing the *AQP9* gene could inhibit the activation of the ERK1/2 signaling pathway, thereby attenuating the inflammatory response in AMI rats, inhibiting apoptosis of cardiomyocytes, and improving cardiac function [34]. This suggests that *AQP9* plays an essential role in the pathogenesis of AMI. Consistent with this study, our study found a statistically significant comparison of *AQP9* subgroups in the combined dataset. Furthermore, the *ROC* curve of *AQP9* in the validation dataset showed that it has a very high diagnostic value for AMI (*AUC* = 0.74). Therefore, we concluded that *AQP9* is a very effective genetic marker for diagnosing AMI.

IL-1 induces the synthesis and expression of various secondary inflammatory cytokines, including IL-6. IL-1b, the primary form of circulating IL-1, is activated by caspase-1 cleavage in the presence of nucleotide-binding oligomerization domain-like receptor family pyridine-containing domain 3 (NLRP3), which is synthesized as the initial precursor [35]. A study concluded that MCC950, a specific NLRP3 inhibitor, could effectively inhibit the release of inflammatory factors IL-1B and IL-1β, thereby suppressing the early inflammatory response after AMI, alleviating fibrosis, and improving cardiac function in mouse model hearts [36, 37]. In our study, *IL1B* was statistically significant in the group comparison between MI and NORMAL groups. Therefore, it is reasonable to assume that *IL1B* may play an essential role in the disease progression of AMI.

*IL1RN* acts as a natural inhibitor of IL-1 by binding to the IL-1R1 receptor, thereby blocking IL-1 signaling [38]. A study reported that tH3 of the gene (IL1RN) encoding IL-1Ra, a natural inhibitor of the pro-inflammatory cytokine IL-1, was associated with an increased risk of myocardial infarction [39]. Interleukin-1 plays a significant role in the pathogenesis of myocardial infarction [40]. We also found a high diagnostic value of *IL1RN* for the diagnosis of MI by the *ROC* curve of the validation group (*AUC* = 0.793). Therefore, we concluded that IL1RN is a valid biomarker for diagnosing AMI.

In addition, our study predicted target miRNAs and lncRNAs for *AQP9*, *IL1B*, and *IL1RN* and constructed ceRNA networks using Cytoscape. This can reveal the upstream regulatory mechanisms of genes at the transcriptome level. Among the target miRNAs of *AQP9*, *IL1B*, and *IL1RN*, the expression of miR-330-3p was associated with plaque rupture of MI [41]. In addition, it has been claimed that *FSTL3* is related to the sensitivity of the heart to ischemic injury. Therefore, we suggest that *FSTL3-miR-330-3p-IL1B/IL1RN* may be a potential RNA regulatory pathway regulating disease progression in MI. In addition, *ACSL4* has been reported in studies related to AMI [42], while miR-590-5p has been shown to inhibit pathological hypertrophy-mediated heart failure [43]. Therefore, we suggest that *ACSL4-miR-590-5p-IL1B* has an essential regulatory role in MI. Of course, the sample size analyzed in our study was relatively small; therefore, future investigators need to increase the sample size and conduct prospective cohort studies to confirm our view further.

## Conclusions

Our study screened three genes (*AQP9*, *IL1B*, and *IL1RN*) that may be potential genetic markers for MI’s early diagnosis and treatment. In addition, we suggest that *FSTL3-miR-330-3p-IL1B/IL1RN* and *ACSL4-miR-590-5p-IL1B* may be possible RNA regulatory pathways to control AMI disease progression.

## Supporting information

S1 Dataset (RAR)
